# The Limits of Conventional Justification: Inductive Risk and Industry Bias Beyond Conventionalism

**DOI:** 10.3389/frma.2020.599506

**Published:** 2020-12-14

**Authors:** Miguel Ohnesorge

**Affiliations:** Department of History and Philosophy of Science, University of Cambridge, Cambridge, United Kingdom

**Keywords:** values in science, conventionalism, methodological conventions, inductive risk, randomized controlled trials, industry bias

## Abstract

This article develops a constructive criticism of methodological conventionalism. Methodological conventionalism asserts that standards of inductive risk ought to be justified in virtue of their ability to facilitate coordination in a research community. On that view, industry bias occurs when conventional methodological standards are violated to foster industry preferences. The underlying account of scientific conventionality, however, is insufficient for theoretical and practical reasons. Conventions may be justified in virtue of their coordinative functions, but often qualify for posterior empirical criticism as research advances. Accordingly, industry bias does not only threaten existing conventions but may impede their empirically warranted improvement if they align with industry preferences. My empiricist account of standards of inductive risk avoids such a problem by asserting that conventional justification can be pragmatically warranted but has, in principle, only a provisional status. Methodological conventions, therefore, should not only be defended from preference-based infringements of their coordinative function but ought to be subjected to empirical criticism.

## Introduction

In 2018, more than 68% of all R&D funding in the United Kingdom stemmed from private donors (National Office of Statistics 2020), a number that still appears moderate when compared to 76% in China and 78% in South-Korea (Eurostats 2019). As Bennett Holman and Kevin Elliot rightly note in a recent meta-review, philosophers have overwhelmingly taken this prevalence of industry funding to be worrisome. It has been argued recurrently that industry funding causes epistemically detrimental “industry bias” across various fields of scientific research (Holman and Elliott 2018, 2). Miriam Solomon, echoing the *British Medical Journal* and *Institute of Medicine*, has recently warned that industry bias poses “the greatest known systematic threat to the objectivity of medical research” (Lo and Field, 2009; Moynihan et al., 2019; Solomon 2020, 439).

However, such strong normative claims require robust epistemological grounds. Surely, industry funded research produces different results, i.e., such that are useful for the respective industry actors. But for what reasons exactly are we holding these differences to be epistemically detrimental? For external interests to qualify as epistemic threats, we ought to have strong reasons that the epistemic standards of our research would be higher without them. The appeal to any such standard, however, sits unwell with recent philosophical claims about the prevalence of so-called inductive risks in various internal stages of scientific research. Inductive risk, for now, can be defined as the risk of “wrongly accepting or rejecting a hypothesis on the basis of evidence” (Biddle 2016). Inductive-risk judgements, so the usual story goes, are non-epistemic value judgements about research design, conduct, and communication. If non-epistemic considerations are so ubiquitous in scientific practice, how can we identify epistemic (as opposed to ethical) standards on which “industry bias” infringes?

Torsten Wilholt has developed a conventionalist solution to this problem, which he later embedded in a position called *methodological conventionalism*. For a methodological conventionalist, conventional standards of inductive risk are epistemically justified due to their ability of facilitating coordination in a scientific community, thereby improving the collective pursuit of knowledge. In other words, sharing conventional methodologies, which implicitly determine standards of inductive risk, is necessary for scientists to engage in coordinated inquiry. Industry bias, on such an account, may be described as the infringement of such conventions for increasing the likelihood of a result preferred by industry funders. Wilholt’s conventionalism has received a warm reception in recent literature on industry funded science and inductive risk.^1^

I will argue, however, that it faces two interlinked problems. First, the conventionalist concept of bias is not able to account for one of the most central epistemic flaws of industry funded research: empirically ineffective conventions. On my reading, this practical problem results from a second, theoretical weakness of Wilholt’s position. The role of conventional justification in science is not characterized sufficiently through appeals to coordination. Although research methodologies can be justified in virtue of their coordinative function, scientists should subject conventional choices to posterior empirical criticism. Based on this insight, I propose a *permissive empiricism*. For a permissive empiricist, the conventional justification of standards of inductive risk can be permitted in light of contextual constraints but is always provisional, i.e., it ought to be substituted by empirical justification at a later point of inquiry. My account thereby preserves Wilholt’s notion of industry bias, while, permitting empirical criticism of *structural industry bias*. The latter results from the institution or perpetuation of empirically ineffective conventions that serve industry interests.

The plan is as follows. First, I reconstruct Wilholt’s conventionalist account of industry bias and its motivation in the argument from inductive risk (AIR). Second, I review whether his position, methodological conventionalism, is well motivated in light of recent work on AIR. Third, I use the case of pharmaceutical trials to show how methodological conventionalists are unable to adequately assess structurally flawed conventions. I close by proposing an empiricist account of standards of inductive risks which preserves Wilholt’s definition of bias while allowing for the empirical criticism of flawed conventions.

## Wilholt on Inductive Risk and Industry Bias

“Industry bias,” roughly, describes epistemically detrimental effects that industry preferences have on the conduct of scientific research. However, as “bias” is certainly a polysemic concept (Resnik 2000), it is necessary to distinguish between (at least) two different senses in which it is used in recent philosophical literature. In a broad sense, industry bias subsumes all factors that increase the likelihood of research to produce results preferable to its industry funders. As such, even the intentional spreading of misinformation in a research community or the outright fabrication of results display instances of so-called “intransigent bias” (Holman and Bruner 2015). In a narrower sense, “industry bias” operates more subtly, as biased researchers select research designs, data interpretations, and ways of communicating that are more likely to produce outcomes preferable to their industry funders. Indeed, statistical findings on biomedical and chemical studies indicate that industry funded studies are significantly more likely to obtain results that serve the interests of their funders (Davidson 1986; Barnes and Bero 1996; Barnes et al., 2006; Schott et al., 2010; Volz and Elliott 2012; Lundh et al., 2017). It is this latter form of industry bias which will be my concern in what follows.

In an influential article, Torsten Wilholt has argued that the argument from inductive risk (AIR) challenges the epistemically detrimental nature of industry bias (Wilholt 2009). Discussions of inductive risk, popularized by Richard Rudner and Carl Hempel, are based on the assumption that the choice of a level of evidential confirmation for accepting a hypothesis is epistemically underdetermined (Rudner 1953; Hempel 1965).^2^ The threshold of evidence that scientists accept as sufficient for a claim to be confirmed displays the risk of possible inductive error that they are willing to take. In making a risk-judgement, researchers face a trade-off between the risk of accepting a claim that is in fact false (false positive) and the risk of rejecting a hypothesis that is in fact true (false negative). Based on Carl Hempel’s account, Heather Douglas has advocated what is usually taken to be the strongest version of AIR, which holds that if false-negatives or false-positives entail non-epistemic consequences, scientists must base their risk-judgements on non-epistemic values. Moreover, Douglas argues, experiments are permeated by such decisions about inductive risk at multiple internal stages, namely when scientists make methodological choices about statistical significance, qualitatively characterize their evidence, and interpret their results. In all these steps, researchers ultimately have to make epistemically underdetermined decisions for which they ought to estimate the consequences of potential false positives and false negatives based on non-epistemic values (Douglas 2000, 577–578).

Whether Douglas’s version of AIR in fact offers a prescriptive or solely a descriptive claim need not concern us further here, as it is the descriptive part of her argument that motivates Wilholt’s risk conventionalist account of industry bias (Wilholt 2009, 94–95). He discusses two case-studies of inductive risk decisions, which would typically be characterized as instances of industry bias:

- Bisphenol A, which is used as a monomer in polycarbonate plastic and has toxic effects due to its similarity to human estrogen, was shown to be carcinogenic in many government-funded studies. However, industry funded experiments were continuously conducted on a strain of laboratory rats known to be insensitive to estrogen, effectively establishing the non-toxicity of bisphenol A (vom Saal and Hughes 2005).
- Exposure to vinyl chloride, likewise used in the production of polycarbonate plastics, is correlated with a rise in mortality rates and a significant increase in liver and brain cancer risk. The Chemical Manufacturers' Association funded various studies based on which the legal regulation of vinyl chloride was prevented. In one of those studies, the famous British epidemiologist Richard Doll dismissed a previous review that claimed to have shown that exposure to vinyl chloride is correlated to liver and brain cancer, and would therefore raise the risk of mortality significantly. Doll argued that all brain cancer cases must be excluded from the mortality risk calculation, as the link between vinyl chloride and brain cancer was only postulated in the very same study, thus having not gone through independent testing (Sass et al., 2005).

Given that AIR holds, Wilholt’s argument goes, industry biases in research on bisphenol A and vinyl chloride may be re-interpreted as potentially justified non-epistemic value judgements about an appropriate level of inductive risk. Both substances, industry researchers might argue, have important applications, as they are widely used to produce polymers for the manufacture of pipes, medical instruments, plastic wraps or wall covers. Non-industry researchers, thus, may in turn be accused of accepting an unduly high risk of false positives. Therefore, the supposedly biased laboratory rat selection and unusually high demand for robustness could be justified in recourse to possible non-epistemic consequences. A similar reasoning, Wilholt shows, can be applied to justify substandard pharmaceutical trials in which drugs are tested against placebos instead of their most effective alternatives (Wilholt 2009, 93).

Surely, there are many *ethical* objections to be raised against these non-epistemic arguments. Cancer risk may be agreed upon to carry more ethical weight than a shortage of plastic wraps or the use of costlier alternative polymers in medical instrument manufacturing (Chiellini et al., 2013). Likewise, we might agree that “public risks” are to be taken more seriously than producer risks (Biddle and Leuschner 2015). However, given that AIR holds, we seem to be left with the conclusion that there is no *epistemic* objection to the supposedly biased research on bisphenol A and vinyl chloride.

However, both findings were not only overturned by the toxicological research community but were deemed to be instances of epistemic failure. Thus, toxicologists invoked a methodological standard that was infringed by the risk decisions in question. Given the considerations above, such apparent standards, and the levels of inductive risk they implicitly or explicitly determine, need an epistemic justification. While Wilholt thinks that there is such a justification, he claims it cannot be grounded in classical individualist epistemology. Rather, it emerges from the *social* epistemology of scientific research. While Wilholt concedes to AIR that we have no purely epistemic reasons to select one specific balance of risk, he contests that methodological standards are nonetheless needed to facilitate the trust of researchers in each other’s results. In turn, trust is *epistemically* warranted because it is required for collective empirical success. In other words, settling on a standard is a “problem of coordination” (Wilholt 2013, 233). Problems of coordination are not solved by empirical evidence or individual rationality but settled by the establishment of a conventional equilibrium between the conflicting utilities of agents. Even if the exact shape of that equilibrium (i.e., the exact balance of inductive risk) cannot be determined rationally, settling on *some* equilibrium maximizes the utilities of all epistemic agents involved. Thus, following conventional methodologies is in the interest of all researchers, as it facilitates mutual trust. As Wilholt’s argument offers a trust-based justification of the standards of inductive risk inherent in conventional research methodologies, he called his view *methodological conventionalism*. For a methodological conventionalist, industry bias can be defined (and criticized) as the “the infringement of an explicit or implicit *conventional* standard of the respective research community in order to increase the likelihood of arriving at a preferred result” (Wilholt 2009, 99).

More recently, Wilholt has presented a more sophisticated version of this argument that distinguishes between conventions simpliciter and epistemic trust. Scientists rely on conventional methodologies to avoid constant deliberations about such implicit risk decisions as highlighted by Douglas. However, even standardized methodologies leave leeway for active value-judgementsto be taken by researchers. In such cases, “reliance presupposes much more than just that other scientists work dependably and professionally in keeping with the rules of the trade. It presupposes that they have the right attitude toward what they are doing—an attitude whose absence might be considered not just regrettable but to a certain degree blameworthy.” (Wilholt 2013, 249)

Wilholt’s modified view suggests that epistemic trust characterizes a stronger kind of reliance that extends beyond implicit standards to include active value judgements. In both versions of his argument, however, the underlying logic remains a conventionalist one. When considerations about non-epistemic consequences enter methodological decision processes, conventional standards or attitudes are needed to fix *some* equilibrium in the trade-off between the risks of false positive and false negatives. As Douglas shows convincingly that such considerations cannot be fully eliminated in scientific research, social coordination can only be achieved if researchers follow methodological conventions.

## Is Methodological Conventionalism Well Motivated?

So far, I have shown that methodological conventionalism is motivated by the prevalence of non-epistemic risk decisions in scientific research. Before discussing the conventionalist account of bias, I will review this motivation in light of recent responses to AIR. In principle, there are two ways of avoiding the conclusion that the ubiquity of inductive risks in scientific research warrant non-epistemic value judgements. One may either show that 1) there is a non-conventional justification of a certain standard of inductive risk or 2) that inductive risk decisions can be sufficiently avoided by scientists.

Strategy 1) is usually based on Bayesian considerations. Wilholt himself is concerned with this, discussing Isaac Levi’s objection to the mid-20th century variant of AIR (Levi 1962). Levi argued that to have a long run approximation of the value of our priors toward *de facto* truth (Pr [H] = 1) or falsity (Pr [**¬**H] = 1) of hypotheses, there is a purely epistemic demand to have a fixed identical threshold *L* for the acceptance of the truth or falsity of a claim, outlawing any trade-offs. However, this oversimplifies the aims of scientific activities, as researchers are not simply looking for true claims. Indeed, there is a nearly infinite amount of arbitrary truths that are scientifically uninteresting. Instead, they are looking for true claims that are significant relative to broader epistemic goals. Even in “basic research” such epistemic significance is indicated by certain epistemic values such as fruitfulness, explanatory scope, or predictive accuracy. Between different epistemic aims or values, however, there can exist context-dependent or systematic trade-offs, casting doubt on the practical realizability of a generally fixed *L*-value (Kuhn 1977; Longino 1996, 44). Instead, an ideal researcher can, at best, follow a utility matrix that prioritizes communicating the truth (i.e., genuinely true or genuinely false claims) over refusing communication or miscommunicating. Such a “weak” commitment to truth, however, leaves leeway to significant trade-offs and consequently does not serve to rationally set appropriate standards of inductive risk.

After Wilholt’s first conventionalist proposal, however, philosophers have scrutinized whether inductive risk might not be sufficiently avoidable after all, thus opting for strategy 2). Such arguments defend the operability of what has been called the value-free ideal (VFI). VFI can be defined as the demand that “the justification of scientific findings should not be based on non-epistemic (e.g., moral or political) values” (Betz 2013, 208). If the operability of VFI can be rescued, one might think, the motivation of risk conventionalism collapses, as any industry preferences can be identified as non-epistemic intrusions.

The most influential recent argument for VFI was put forward by Gregor Betz, explicitly targeting, among other views, Wilholt’s adherence to a variant of AIR (Betz 2013, 208). While conceding that the inductive risk in making binary judgements about accepting or rejecting a hypothesis cannot be eliminated, he denies that scientists must make judgements of that form. Instead of acceptance/rejection assertions, scientists can qualify their results by stating all instances of inductive risk in their research. For example, they may hedge their claims by elucidating the statistical certainty or significance of their findings (e.g., *x* is correct “with a certainty of 95%” or x is significant “to a *p*-value of 0.04”). Likewise, they may modalize or conditionalize their conclusions more generally (e.g., “it is possible/unlikely/plausible” that x is correct or “if we admit a possible error of *y*/a model organism *z,*” *x* is correct). While one may contest that every hedged claim comes with second-order risk as “probabilistic hypotheses are just as open to inductive risks as others” (Brown 2013, 834; Douglas 2009, 85), it is not clear whether this actually poses a threat for Betz’s defense of the VFI. His argument does not aim to show that inductive claims can be completely free of risk, but qualified “beyond reasonable doubt.” If he is correct about this, scientists may avoid the impact of non-epistemic considerations on their results by acknowledging and stating the risks of their inductive inferences in form of probabilistic, modal, or conditional claims. Contra Wilholt, industry bias may, on this account, be identified as epistemically detrimental if it inhibits the proper communication and/or minimization of inductive risks (Betz 2013, 216).

For Betz’s proposal to have any bearing on Wilholt’s use of AIR, it needs to be shown that it can in fact be operationalized successfully to avoid value-judgements and thus offers an operable epistemic standard against which industry biases can be assessed. However, the validity of Betz’s own example for value freedom in action, the *Guidance Note for a consistent treatment of uncertainties* by the Intergovernmental Panel on Climate Change (IPCC) (Mastrandrea et al., 2011), has been strongly contested. The Guidance Note tries to map inductive risk based on “states of scientific understanding,” by reference to which scientists involved in a global evaluation of climate research are supposed to qualify their findings. However, as Katie Steele has argued based on an older IPCC report, such confidence scales (e.g., range of 1–10, intervals of 1) are too coarse-grained to properly communicate inductive risks. This situation is complicated further by the underdetermined classifying of some of those intervals as displaying “high,” low,” or “medium” confidence (Steele 2012). The problem of applying Betz’s hedging principle is not limited to the case considered by Steele, but persist within the newer IPCC reports (Steel 2016; Frisch 2020). Moreover, Stephen John has shown that the IPCC even commits to inductive risk judgements by including or excluding peer-reviewed papers. In its fourth report, scientists refrained from making a prediction regarding the melting of the West Antarctic Ice Shield because they excluded a study not yet gone through peer-review (O’Reilly et al., 2012; John 2015, 7).

Beyond its lack of applicability to the IPCC-case, however, John argues that a modest version of the VFI (VFI_modest_ in what follows) can be rescued from Betz’s proposal. It seems that the idea of hedging claims is a fruitful one in principle, as it decreases inductive risks. He proposes that hedging claims “beyond reasonable doubt” is enough to consolidate VFI_modest_, even if it is less reliable in eliminating non-epistemic value judgements than Betz concedes. Reformulating Duncan Pritchard’s epistemic-safety definition of knowledge, John argues that “knowledge” itself can be roughly defined as our body of claims that are true beyond reasonable doubt. If scientists hedge their claims beyond reasonable doubt, they thus pursue a purely epistemic aim, namely genuine knowledge (Pritchard 2005; John 2015, 163). One problem for VFI_modest_ approximates canonical issues around Popperian testability. The history of science gives evidence that proliferating doubtful hypotheses, contrary to norms of epistemic caution, can be epistemically beneficial in the long run (Lakatos 1999; Feyerabend 2002; Chang 2014, ch.2). Thus, scientists have, in certain cases, purely epistemic reasons for not following VFI_modest_. Beyond such broader issues, VFI_modest_ certainly does not offer an epistemic standard strong enough to criticize the cases of industry bias that Wilholt discusses. Richard Doll dismissed the health hazards of vinyl chloride precisely by appealing to epistemic caution (Wilholt 2009, 93). Thus, the adherent of VFI_modest_ will continue to be puzzled by the actions of the toxicological community which not only overturned Doll’s research findings, but retrospectively deemed their initial acceptance an instance of epistemic failure (Sass et al., 2005).

## The Problem of Flawed Conventions

I have argued that the motivation of methodological conventionalism by appeal to inductive risk decisions in various stages of scientific research is robust in light of recent criticism of AIR. Remember that Wilholt further intends his position to justify epistemic criticisms of industry bias as “the infringement of an explicit or implicit *conventional* standard of the respective research community in order to increase the likelihood of arriving at a preferred result” (Wilholt 2009, 99). Some of the most strongly voiced criticism of industry influence on scientific research, however, does not target infringements on methodological conventions, but explicitly points to flaws *in* such conventions. For example, a recent collaborative article in the British Medical Journal states:

“Sponsoring companies have obvious financial incentives to overstate product benefits and downplay harms. But these incentives are enabled by our imperfect methods of evaluation, which can be exploited in myriad ways, consciously or unconsciously, at all stages of the process.” (Moynihan et al., 2019, 2).

To better understand the implications of flawed standards for methodological conventionalism, let us look at an illustrative example: pharmaceutical drug trials involving human subjects. As Jacob Stegenga has argued at length, the current organization of the system of randomized controlled drug trials (RCTs) hinders the detection of harms. RCT testing is split up in three separate phases (P_1_-P_3_). Only drugs that were successful (i.e., harm-free) in P_1_ successively enter P_2_ and P_3_ trials. However, an estimated 95% of P_1_ results remain unpublished by the pharmaceutical companies owning the studies’ publishing rights (Decullier et al., 2009). Thereby, Stegenga argues, relevant evidence about the harmfulness of the tested molecules, the broader classes of molecule they belong to, as well as medical drugs overall is lost:

“Any tested molecule x is a member of the class of molecules of type T, and this class is itself a member of the class of all drugs D. Evidence from a phase 1 trial on x is relevant, obviously, to the harm profile of x, but is also relevant to the harm profile of T (albeit more indirectly), and is also relevant to the harm profile of D (more indirectly still).” (Stegenga 2018, 138).

The unavailability of a majority of the evidence about the harms of *x*, *T*, and *D* constrains the reference class based on which the prior probabilities for harmful effects in future drug trials are determined. More formally: the conditional probability Pr(K|E) of *x* being harmful in a future P_1_, P_2_, or P_3_ trial, where K is the hypothesis that *x* is harmful and *E* the relevant new evidence, will always be unduly low due to the constrained reference class of K. Moreover, due to the constrained evidence about the harm profile of *T* and *D*, the value of prior probabilities in future trials involving molecules of the same class as *x*, and, in a less significant manner, any other molecule, will decrease. Overall, the prevalence of harms of any specific drug and of pharmaceuticals in general can therefore be expected to be way higher were it not for the withholding of evidence from P_1_ trials (Stegenga 2018, 138–139). Stegenga identifies an apparent trade-off between two forms of statistical power, where “statistical power” refers to “the sensitivity of a trial to detect an effect of the intervention under investigation, when there is such an effect to be detected” (Stegenga 2018, 141). Statistical power is a function of a trial’s effect size, the number of subjects under investigations, and the variability of the data. In the case of P_1_, P_2_, and P_3_ statistical trials, the power_H_ to detect harms partially trades-off with the power_B_ to detect benefits of drugs.

It is possible to directly connect the RCT case to Wilholt’s AIR-based line of reasoning. In fact, one can easily recast the choice of a balance between the two types of statistical power as a choice of a standard of inductive risk. If power_H_ increases, we face a higher risk of false positives (harmless drugs that are wrongly assessed to be harmful), if power_B_ increases, we face a higher risk of false negatives (harmful drugs that were wrongly assessed to be harmless). Now, with a bit of counterfactual reasoning, it is possible criticize the empirical performance of the current standard of inductive risk while avoiding an ethical judgment about the appropriate balance of risks. Consider the possibility of publishing more P_1_ results. As a consequence, the absolute power to detect harms in RCT system will increase *without* decreasing the absolute power to detect benefits. While the *relative* balance between power_H_ and power_B_ tips toward power_H_, our methodological decision would not decrease the ability of pharmaceutical drug trials to detect beneficial drugs. The changes in the standard of inductive risk are not the result of weighing ethical consequences, nor solely of changing coordinative conventions. If all P_1_ results were to be published, we would improve the *empirical* performance of the research methodology in question. The currently operative conventional standard of risk in the pharmaceutical RCTs, it follows, has been *empirically ineffective*.

Now, methodological conventionalism only licenses epistemic criticism of those preference-based decisions that infringe on conventional standards of inductive risk. The testing for harms by pharmaceutical companies, however, does not infringe on the current conventions but exploits and perpetuates their inherent flaws. As a consequence, it does not qualify as being biased in the way defined earlier. In fact, Wilholt admits “that it might be claimed that sometimes the conventional standards of a research community are themselves distorted by interests and preferences in an epistemologically problematic way” (Wilholt 2009, 2). If all that would be at stake is the adequate domain for the definition of bias as infringements on conventions, such a disclaimer might suffice to avoid the problem. We could simply exempt flawed conventions from our definition of industry bias and treat them as a separate kind of epistemic problem. Methodological conventionalism, however, goes beyond a conventional concept of bias, as it aims to offer a general account of the justification of standards of inductive risk. The RCT case offers a counterexample to such a view as it illustrates that inductive risk equilibria that are set by conventionally accepted methodologies can be epistemically criticized beyond their ability to facilitate coordination. As it stands, conventionalists appear to be unable of accounting for the purely empirical target of such criticism even if we (quite artificially) separate them from the problem of industry bias.

Wilholt himself seems aware of the problem, as he offers a reworked account of conventionality in a 2016 paper, which appears to be more promising regarding its ability to deal with the problem of flawed conventions (Wilholt 2016). There, he argues that discussions of AIR have unduly neglected the rate at which varying methodological conventions lead researchers to empirical results. While inductive risk judgements are generally taken to involve a trade-off between the reliabilities of negative and positive results, Wilholt now takes them to involve an additional third dimension (see also: Steel 2016). He characterized the latter as a method’s “power”, defined as the rate at which it “generates definitive results, given a certain amount of effort” (Wilholt 2016, 227) Hence, what is desirable in a method of inquiry (from an epistemic perspective) can only be captured by considering all three dimensions: the reliability of positive results, the reliability of negative results, and the method’s power. For each method, these three magnitudes form a triple that I will call the inquiry’s distribution of inductive risks (Wilholt 2016, 227).

Thus, the adoption of methodological standards is not solely coordinating the risks involved in positive and negative reliability but is likewise constrained by its function in delivering new findings. Could a methodological conventionalist, then, account for cases like the RCT system by invoking changes in such three-dimensional *distributions of inductive risks*? I do not think so. While Wilholt’s focus on inquiries' absolute power correctly acknowledges that different conventional standards of inductive risk do vary in their empirical effectiveness, he does not concede that the latter can be improved without sacrificing the reliability of positive or negative results:

“The three dimensions of the vector are antagonistic to each other in the sense that each of them alone can easily be increased at the cost of one or both of the others, so that any methodological choice involves a trade-off between the three dimensions.” (Wilholt 2016, 228) (my italics).

In light of the above discussion, the claim that “any methodological choice” trades-off against positive and negative reliability of standards of methodologies seems incorrect. In fact, it appears to be possible to *increase* the RCT system’s ability to 1) deliver empirical results and 2) to avoid false negatives, without thereby *decreasing* its ability to avoid false positives. By not acknowledging the possibility of such empirical improvements, methodological conventionalism fails to license a robust criticism of empirically ineffective conventions and so does not offer sufficient grounds to discuss the epistemic dangers of industry funded science.

## Conventions and Empirical Criticism

The problem of flawed conventions indicates the shortcomings of methodological conventionalism. As an account of the justification of methodologies choices, it does not license criticisms of purely empirical (as opposed to coordinative) flaws in conventionally justified standards of inductive risk. In what follows, I want to propose an alternative account, dubbed *permissive empiricism.* My proposal constructively departs from methodological conventionalism by introducing a more sophisticated account of conventionality in science. In doing so, I aim to preserve the merits of Wilholt’s focus on the role of conventions in discussing inductive risk decisions and identifying industry bias. In fact, I hope that the position that I will develop can offer a two-fold constructive *improvement* on methodological conventionalism’s theoretical and practical weaknesses. Theoretically, I hope to offer a more sophisticated analysis of conventionality that aligns Wilholt’s insights with an uncontroversial empiricism. Practically, my position should offer a more powerful framework to identify instances of industry bias.

Recall that Wilholt understands the choice of standards of inductive risk as a “problem of coordination” (Wilholt 2013, 233). If scientists do not trust each other to take similar risks in their research, the research community’s coordination suffers, which negatively impacts its overall epistemic success. Thus, it is epistemically warranted that the risk decisions of “information producers […] and information users [in the research community] are (approximately) the same” (Wilholt 2013, 248). As the RCT case in section four shows, this leaves the balance of inductive risk 1) a matter of convention, and, therefore, 2) not liable to direct empirical criticism. Although I agree with Wilholt that methodological standards of inductive risk can be justified in virtue of their conventional function in facilitating coordination, I will argue that 2) does not follow, as conventions are not immune to empirical criticism. In fact, scientist *ought* to aim at providing such criticism to avoid perpetuating empirically ineffective conventions.

The long history of conventionality in the philosophy of science offers a good starting point to understand how standards of inductive risk can be subjected to empirical criticism. Ernst Mach originally introduced the problem of coordination in a discussion of thermometric intervals. As thermometric intervals are given as a function of the expansion rate of a thermoscopic substance, he argued, we ought to choose a substance expanding as uniformly with increasing temperature as possible. However, “uniformity of expansion” presumes the thermometric scale we want to define, given that we have no scale-independent possibility of operationalizing “temperature”. Thus, we face an undetermined decision between different standards of measurement which will all fulfill a conventional purpose of facilitating coordination (Mach and McGuiness, 1986, 52). Such problems seem to require different responses than the decisions typically faced by scientists. Generally, we would want scientists to choose explanations, theories, or methods based on some form of available or expected empirical evidence. When mid-nineteenth century physicists chose whether to base the thermometric intervals on the expansion rates of mercury, air, or alcohol, however, they had no reason to think that any one of those would perform better empirically, i.e., record more accurately the changes in absolute temperature.^3^ If chosen as standard, each thermoscopic substance would simplify certain kinds of measurement situations while making others more complicated. By side-stepping this underdetermined choice and agreeing on *some* standard, however, physicists could establish an equilibrium between those partially conflicting utilities.^4^ Thereby, they did not settle conclusively the scientific problem of mapping “temperature” onto the physical world, but epistemically improved the social pursuit of temperature research.

Acknowledging that some aspects scientific practice involve conventionality in the above sense, however, is widely accepted and does not yet ground a conventional*ism*. As David Lewis points out, conventional*ism* entails some additional beliefs about the *power* of conventions. Thus, subscribing to a conventionalism about a goal-oriented social practice *x*, expresses a view about the extent to which the organization of x is settled by coordinative equilibria–as opposed to appeals to empirical evidence (Lewis 1969, 4). Thus, if we agree with Wilholt that scientists can avoid making inductive risk decisions by following methodological conventions, it remains open how far-reaching the implications of that insight are. Flawed standards as in the RCT-case discussed above, in particular, force us to evaluate the degree to which standards of inductive risk remain a matter of social coordination and to which degree the methodologies by which they are entailed can be criticized empirically. Here, a return to the well-studied role of conventionality in thermometry proves insightful. As Hasok Chang notes in his canonical study on the subject, thermoscopic substances were only chosen for conventional reasons *initially*, yet meticulously tested on variations against each other afterward (Chang 2004, 59). That is, although first attempts at standardization were based on conventional decisions, thermometric standards could eventually be subjected to comparative empirical scrutiny. In the long run, the relative performance of alternative ways of standardizing temperature (i.e. in reference to different thermoscopic substances) could be compared based on the substances’ relative performance. While choices of measurement standards were thus based on both their conventional ability to facilitate coordination and their empirical success, the *power* of conventionality in such choices *decreased over time*. Thus, a conventional judgment about the fixation of a coordinative equilibrium successively made room for a growing body of empirical evidence.

Now recall the case of harm detection in RCTs. On the conventionalist account, a standardization of inductive risk, i.e. a fixed balance between power_H_ and power_B_, is epistemically warranted and the methodological choices constituting that standard can be justified in virtue of their conventionality. However, I take Stegenga’s analysis to show that a modification of the current methodological conventions promises to be epistemically beneficial. Thus, if we require the publishing of all P_1_ results, thereby modifying the standard of risk, we can expect more empirical success in the actual detection of harms without decreasing the trials' capacity to detect benefits. In line with the temperature analogy above, the performance of the current standard can be epistemically assessed based on its comparative empirical success. Of course, there is a crucial difference between the two examples. In the case of thermometry, physicists were able to conduct a comparative evaluation of alternative conventions based on their actual empirical success, whereas epistemic criticism of the current RCT system is based on counterfactual reasoning. Both, however, indicate that strongly conventional methodological decisions can, be subjected to empirical criticism, at a later stage. Such criticism does not consist of a weighing of inductive risks, but of an analysis of the empirical performance of the operative methodological conventions that set the respective standards of risk.

Given these examples as well as Lewis’s distinction between conventionality and conventional*ism*, my worry about the unwarranted implications of Wilholt’s proposal can be stated more succinctly. In scientific practice, conventionally justified methodological choices often qualify for empirical criticism (or justification) at a later stage of research. If scientists can limit conventionality by extending the scope of empirical scrutiny, they *ought* to do so. While Wilholt’s notion of bias as preference-based infringements on methodological conventions offers an important epistemological criticism, caution is needed before generalizing it into an “-ism” of any sort. Standards of inductive risks that are determined by conventional methodologies are epistemically necessary and should be defended against preference-based infringements. However, pace Wilholt, coordination offers merely a *preliminary* form of justification, which should be substituted by empirical arguments wherever possible. In theory, such arguments may consist of counterfactual criticism (as offered by Stegenga in the RCT case), or even become actualized in comparisons of empirical performance (as in the thermometry example).

## Toward a Permissive Empiricism

I have argued that methodological conventionalism cannot adequately address the problem of flawed methodological standards and even runs the risk of providing them with a justification in terms of their conventional utility. I have traced the cause of that problem to an insufficient analysis of scientific conventionality. While scientists avoid inductive risk decisions by following methodologies that are not justified empirically but in terms of their coordinative function, such conventional justifications are merely preliminary. In scientific practice, conventional methodological choices can be subjected to posterior criticism by comparing their respective empirical performance. In such processes, conventional justification is substituted by empirical justification. By stressing that such substitutions are warranted, we can avoid the problem of flawed conventions.

Given these qualifications, we can preserve Wilholt’s insights in a straight-forward and, I hope, uncontroversial empiricist position. All the standard empiricist has to concede is that standards of inductive risks can be provisionally justified in virtue of their coordinative function. Such a *proviso* accounts for practical constraints that are an ineliminable element of scientific practice, like a shortage of information about a new domain of inquiry or a lack of financial or instrumental resources. As Wilholt has shown convincingly, socio-epistemic coordination warrants *some* standard. However, conventional justifications should, in principle, be regarded as merely *preliminary*. As such, they ought to be subjected to posterior empirical criticism. To illustrate this in another case, take Wilholt’s own example of toxicological research into the health risks of exposure to bisphenol A. The selection of model organisms for testing bisphenol A, of course, comes with non-epistemic risks due to the potential health hazards that missing legal restrictions would cause to humans. A conventionalist would now object (epistemically) to the preference-based choice of a specific rat strain outside of a conventional class of model organisms. However, we can also make a straight-forward empirical argument about the comparative suitability of the different rat strains available. If the toxicity of bisphenol A is linked to its similarity to estrogen, an estrogen-insensitive rat strain will have smaller relative empirical success in detecting the potential harms of exposure to humans. Thus, not only did industry research infringe on the toxicological conventions, but it negatively impacted their empirical performance. While the former criticism identifies epistemically detrimental effects on the *collective* coordination of inquiry, the latter would show how one *particular* (potential) standard is empirically ineffective. I concede that, in this case, the empirical criticism does not target a methodological convention. I hope, however, that it sufficiently illustrates the relevant difference. Afterall, the same argument applies if the *conventional* class of model organisms (instead of the organism used in a *particular* experiment) would be composed of estrogen-insensitive rats.

In the debates on value-freedom and inductive risk, such a permissive variant of empiricism offers a compromise between methodological conventionalism and the VFI. In line with the former, it asserts that most inductive risk judgements are not made by individual scientists, but are settled implicitly by conventionally justified standards. Thus, conventional justification may only be accepted if ignorance or financial and experimental constrains keep us from testing the relative empirical performance of different standards of inductive risk.^5^ In line with defenders of the VFI, however, permissive empiricism maintains that an effective elimination of non-epistemic risk decisions is generally warranted. This normative aim, however, is not achieved through specific forms of communication, but by means of posterior empirical criticism of conventional standards.

If the reader has followed my arguments this far, she might still find my alternative somewhat less elegant than either the 1) VFI or 2) methodological conventionalism. I concede that both offer a simpler response to the problem of inductive risk and, moreover, a single accompanying strategy to identify epistemically detrimental bias. I hope to have shown, however, that both run into serious difficulties, as they are either 1) practically inoperable or 2) implicitly allow for the justification of flawed standards. Beyond these negative arguments, moreover, the inconvenience introduced by a permissive empiricism is smaller than it might appear on first sight. While it permits not one but two kinds of justifications for standards of inductive risk (conventional and empirical), neither of them is grounded in any particularly uncomfortable or even novel epistemological claim. They simply pay tribute to the fact that scientific inquiry has a social *and* an empirical dimension. As such, research needs both 1) effective organization (i.e., coordination qua conventions) and 2) sensibility to the behavior of its corresponding target-systems (i.e., empirical utility). It should not be too controversial to regard arguments pertaining to 2) as the stronger form of justification or criticism. After all, corresponding to these two dimensions, we found industry influence to have two forms of epistemically detrimental consequences. Financial incentives infringe on the necessary conventions of a field or perpetuate empirically ineffective conventions in that field. The former epistemic danger is captured by industry bias in Wilholt’s sense, while the latter might be dubbed *structural industry bias*, as it is the result of structurally flawed standards.

## Conclusion

I have offered a criticism of methodological conventionalism. Wilholt’s proposal is an important intervention in both the debates on value-freedom and industry bias. Not only does it highlight the neglected role of social conventions in handling inductive risk, but its definition of bias as preference-based infringements of conventional standards licenses criticisms of a crucial epistemic danger in industry funded science. However, I have argued that it suffers from a theoretical and a resulting practical weakness. *Theoretically*, it offers an underdeveloped analysis of scientific conventions, which fails to highlight how scientists are able to eliminate conventionality through posterior empirical criticism. Pure coordination problems that are confronted by settling a conventional equilibrium often become solvable on the basis of empirical evidence at a later stage of research. In such processes, a weaker type of justification and criticism is substituted by a stronger alternative. *Practically*, the neglect of this facet of conventionality makes methodological conventionalism unsuited to deal with what I dubbed the problem of flawed conventions. Some of the epistemically most detrimental consequences of industry preferences are not infringements on conventions, but the institution or perpetuation of empirically ineffective conventions.

In a constructive departure from methodological conventionalism, I tried to offer an account that preserves its insights while including a more qualified notion of conventionality. *Permissive empiricism*, as I dubbed it, is the following two-partite view on the justification of standards of inductive risk. Methodological choices that determine certain balances of inductive risk can be provisionally justified in virtue of their conventional function in setting coordinative equilibria. Such justifications, however, are merely preliminary, as they ought to be substituted by empirical arguments. Thus, if not blocked by financial, experimental, or other constrains, conventions should be evaluated based on their comparative empirical success.

Corresponding to the two kinds of epistemic justification invoked above, my empiricist framework licenses two kinds of criticisms of industry bias. The first has been exhaustively characterized by Wilholt and targets preference-based infringement on conventional standards of inductive risk. I proposed *structural industry bias* as a name for the additional type of bias I introduced. Structural industry bias occurs if industry influence perpetuates or institutes conventional standards that are empirically ineffective. Identifying and criticizing this second kind of bias is crucial for any evaluation of the dangers of industry funded science. As Stegenga’s case against the current RCT-system illustrates, such criticism is not an idle epistemological worry, but has direct relevance for the epistemic integrity of scientific research. Attention to structural industry bias, is thus of central importance for the successful regulation of industry funded research.

## Data Availability Statement

The original contributions presented in the study are included in the article/Supplementary Material, further inquiries can be directed to the corresponding author.

## Author Contributions

The author confirms being the sole contributor of this work and has approved it for publication.

## Funding

This work was supported by the German Academic Scholarship Foundation (award number: 2345990) and the German Academic Exchange Service (award number 57437986).

## Conflict of Interest

The author declares that the research was conducted in the absence of any commercial or financial relationships that could be construed as a potential conflict of interest.

## Footnotes

1 The only criticism may be found in Betz (2013) and is discussed explicitly in section “*Is Methodological Conventionalism Well Motivated?*”.

2 For the statistical origin of inductive risk see: Wald (1942), Churchman (1948).

3 This is, of course, at best an oversimplification of the complicated history of thermometry, which merely serves to illustrate, not to substantiate, Mach’s argument.

4 This framing in terms of equilibria stems from Lewis, (1969).

5 Such as the interpretation of autopsy slides of laboratory rats in dioxin cancer studies discussed in Douglas (2000), 569–570.
